# Stress Makes the Difference: Social Stress and Social Anxiety in Decision-Making Under Uncertainty

**DOI:** 10.3389/fpsyg.2021.578293

**Published:** 2021-02-22

**Authors:** Kristina M. Hengen, Georg W. Alpers

**Affiliations:** Department of Psychology, School of Social Science, University of Mannheim, Mannheim, Germany

**Keywords:** anxiety, stress, decision-making, avoidance, social anxiety

## Abstract

Stress and anxiety can both influence risk-taking in decision-making. While stress typically increases risk-taking, anxiety often leads to risk-averse choices. Few studies have examined both stress and anxiety in a single paradigm to assess risk-averse choices. We therefore set out to examine emotional decision-making under stress in socially anxious participants. In our study, individuals (*N* = 87) high or low in social anxiety completed an expanded variation of the *Balloon Analogue Risk Task* (*BART*). While inflating a balloon to a larger degree is rewarded, a possible explosion leads to (a) a loss of money and (b) it is followed by an emotional picture (i.e., a calm vs. an angry face). To induce stress before this task, participants were told that they would have to deliver a speech. We operationalized risk-taking by the number of pumps during inflation and its functionality by the amount of monetary gain. In addition, response times were recorded as an index of decisional conflict. Without the stressor, high socially anxious compared to low socially anxious participants did not differ in any of the dependent variables. However, under stress, the low socially anxious group took more risk and earned more money, while high socially anxious individuals remained more cautious and did not change their risk-taking under social stress. Overall, high socially anxious individuals made their decisions more hesitantly compared to low socially anxious individuals. Unexpectedly, there were no main effects or interactions with the valence of the emotional faces. This data shows that stress affects socially anxious individuals differently: in low socially anxious individuals stress fosters risk-taking, whereas high socially anxious individuals did not alter their behavior and remained risk-averse. The novel *eBART* is a promising research tool to examine the specific factors that influence decision-making.

## Introduction

There is convincing evidence that stress and anxiety change how we evaluate the risk and benefit of an option and that they strongly influence our decisions (Kudielka et al., 2009; Pittig et al., 2015). They both occupy cognitive resources during information processing (Botvinick et al., 2001) and may hinder adaptive processing of emotional as well as cognitive conflicts which might, for example, result in longer response times (e.g., Etkin and Schatzberg, 2011; Larson et al., 2013). Although they share a similar pattern of physiological reactions (Dickerson and Kemeny, 2004), they differ in the interpretation of the situation (Sarason, 1984). While stress emerges when an organism is confronted with overstraining demands (Koolhaas et al., 2011), anxiety is an emotional consequence of perceived threat (see Rosen and Schulkin, 1998).

Despite their documented relevance, little is known about the specific and mutual effects of stress and anxiety on risk-taking behavior, especially in decisions where approach-avoidance motivations compete against each other. Because anxiety is the most important motivation for avoidance behavior (Hofmann et al., 2008) and stress is common in many situations in daily life (McEwen, 2008), it is of special interest to investigate both states in the context of an approach-avoidance conflict.

Several researchers examined the impact of stress on risk-taking (e.g., Starcke et al., 2008; Kassam et al., 2009). A recent meta-analysis concludes that stress leads to riskier decisions, especially when risk-taking is dysfunctional (Starcke and Brand, 2016). On this basis, we sought to investigate the effects of stress and anxiety on risky decision-making in an ecologically valid paradigm in high and low socially anxious individuals.

To experimentally induce stress in the laboratory, the so-called *public speaking task* is frequently used and has been shown to be an effective stressor is (e.g., Steele and Josephs, 1988). In this task, participants are told that they are to give a speech on a controversial topic without time for preparation. They are also told that they will be videotaped and later evaluated by experts after the experiment. This task is a reliable method to evoke emotionally triggered self-reported and physiological arousal (e.g., Mühlberger et al., 2008; Schulz et al., 2008). Especially in social anxiety, where a classificatory feature is the fear of being embarrassed in front of an audience, this method effectively induces anxiety.

In addition to the stress induction, the selection of the appropriate decision-making paradigm is most relevant. Potential paradigms differ with respect to the (un)certainty of the risk involved in each decision. Many laboratory tasks measure decision-making under uncertainty, where the probability of an outcome and the outcome itself are unknown. For example, being stressed participants learned reward contingencies under uncertainty in the *Iowa Gambling Task (IGT)* more slowly than non-stressed individuals (Preston et al., 2007). Similarly, in the *Game of Dice Task* (*GDT*; Brand et al., 2005), stress interfered with task performance and consequently lead to more disadvantageous decisions (Starcke et al., 2008). Common to these paradigms is that they require participants to learn reward and loss contingencies through feedback during the task. However, reward contingencies in these tasks are not obviously clear and participants are less likely to learn them through feedback. In addition, induced stress may interfere with cognitive resources during information processing and may further deteriorate emotional feedback processing (Starcke et al., 2011). Furthermore, these tasks also encourage participants to focus more on the potential losses because riskier decisions are classified as disadvantageous.

Thus, we made use of a well-established paradigm to assess the shift from decisions under uncertainty to decisions under risk by feedback learning. In the *Balloon Analogue Risk Task* (*BART*; Lejuez et al., 2002), participants inflate a computer-simulated balloon and earn a certain amount of money with each pump. Simultaneously, with each pump, the risk of the balloon exploding increases. If the balloon explodes, the money earned so far is lost. As decision-making in everyday life includes the potential of reward and loss at the same time (Leigh, 1999), this task is an ecologically valid paradigm to model risk-taking under experimental conditions. It transfers well to real-life risk behavior (Lejuez et al., 2003), as taking a risk often includes several sequential decisions and seldom all-or-nothing decisions, as implemented in other decision-making tasks. Indeed, the number of pumps in the *BART* correlates with risk behavior in real life, such as smoking or heavy drinking (Lejuez et al., 2003). It also fosters individuals to focus more on the immediate incentives and it rewards riskier decisions. Many studies illustrate heightened risk-taking and, consequently, more advantageous decisions under stressful than under non-stressful conditions (e.g., Reynolds et al., 2013).

In this line, anxiety is often manipulated as an emotional state for risk-taking behavior in the laboratory. In addition, when perceived in a decision-making situation, anxiety exhibits a powerful influence on our decision-making behavior (Finucane et al., 2000; Pittig et al., 2015; Buelow and Barnhart, 2017). Anxiety in particular leads to biased risk estimations of negative events, especially of negative outcomes (Hengen and Alpers, 2019). From a clinical perspective it is evident that this can lead to maladaptive avoidance behavior, which is a classificatory feature of anxiety disorders (Hofmann et al., 2008). Some researchers argue that this biased risk evaluation is a mediating factor between heightened risk perception and higher risk avoidance (Maner and Schmidt, 2006; Lorian and Grisham, 2010). A systematic study on these fear-driven estimated risks showed heightened risk estimates for negative outcomes of fear-relevant encounters and not of the encounter itself (Hengen and Alpers, 2019). Furthermore, less is clear about the distinct and interacting effects of anxiety and stress on the distinct components of risk perception.

In turn, avoidance behavior is an important factor in the etiology and maintenance of anxiety disorders (Hofmann et al., 2008; Craske et al., 2009) and the reason for a maladaptive decision-making strategy. For example, high socially anxious individuals report they avoid social opportunities (Antony and Stein, 2008) even if they are aware of the incurred costs of their decision (Kashdan et al., 2008). Few laboratory paradigms have replicated this finding, and fewer still that were aimed at modeling this approach-avoidance conflict. We previously used a modified version of the *IGT* (Bechara et al., 1994) and added fear-related stimuli as indicators for advantageous or disadvantageous choices (Pittig et al., 2014a,b,c; Bublatzky et al., 2017). In a study with high socially anxious individuals, they avoided pictures of angry faces at the expense of monetary losses (Pittig et al., 2015). Thus, this modified version of the *IGT* is one of the few paradigms that validly assesses the approach-avoidance conflict in anxiety.

In the previously developed emotional *BART* (*eBART*; Hengen and Alpers, accepted pending revision), we added a fear-relevant event as a second consequence in addition to the risk of losing the money earned after an explosion to create an approach-avoidance conflict. Thus, when participants inflate the virtual balloon, they run the risk a) of losing the money earned with each pump – as in the established BART – and b) being confronted with task-irrelevant but fear-relevant stimuli. We maintained the loss of money from the original version to ensure that non-fearful individuals would not inflate all balloons to the very last pump, which would otherwise be the normative response.

This modified *eBART* proved to be an ecologically valid method in modeling the fear-driven approach-avoidance conflict in spider-fearful individuals. For the present study, we replaced the fear-relevant stimuli spiders with angry (and neutral) faces as fear-relevant outcomes for our socially anxious group. Angry facial expressions were effective in eliciting social threat and rejection in high socially anxious individuals in previous research (e.g., Mogg et al., 2004; Wieser et al., 2009; Pittig et al., 2015). As detailed above, to evoke stress in low socially anxious and anxiety in high socially anxious individuals, we made use of the *public speaking task*.

When we are confronted with such a difficult situation, namely the approach-avoidance conflict in the *eBART*, we are challenged to allocate cognitive resources toward the task’s demands (Botvinick et al., 2001). Anxiety and stress may modulate our capacity to allocate these resources to solve the conflict situation. Whereas anxiety leads to more conflict adaptation and consequently to longer response times (e.g., Clayson and Larson, 2011; van Steenbergen et al., 2015; Hengen and Alpers, accepted pending revision), little is known about the specific effects of stress and the mutual effects of anxiety and stress on response times. Therefore, just as in our recently modified *eBART*, we set out to measure response times.

As it is also unclear if effects found in studies with spider-fearful individuals can be generalized to social anxiety (see Berdica et al., 2018), we sought to replicate our earlier findings of spider-fearful and spider non-fearful individuals (Hengen and Alpers, accepted pending revision) in social anxiety. We expected that social anxiety would lead to an overall risk avoidance behavior; this being most pronounced in the context of fear-relevant stimuli. As the *eBART* rewards risk-taking behavior, anxious individuals compared to non-anxious ones should not learn to adapt their behavior to a more risk-taking and functional strategy. Furthermore, we expected that stress would provoke more risk-taking in non-anxious but not in anxious individuals. To add to previous research and findings on risk perception, we argue that anxiety and stress should result in heightened risk estimates of negative outcomes of fear-relevant encounters, but not in heightened risk estimates of fear-relevant encounters themselves. Furthermore, as stress and anxiety are affective states that limit cognitive resources and impede conflict processing, we expected longer response times for both anxious and non-anxious individuals under stress.

## Materials and Methods

### Participants

A large sample (*N* = 87)^1^ of individuals with an age range of 18–32 (*M* = 22.37, *SD* = 3.32) from the local community and from students at the University of Mannheim were first screened for high and low levels of social anxiety with the mini-*Social Phobia Inventory* (mini-SPIN; Connor et al., 2001). This questionnaire consists of three items that must be rated on a 0 (*not at all*) − 5 (*absolutely*) Likert scale. They capture the key features of social anxiety, namely anxiety of feeling ashamed and the avoidance of social activities [American Psychiatric Association (APA), 2013]. Participants with values ≤2 were classified as low socially anxious and participants with values ≥6 as high socially anxious (Connor et al., 2001; Seeley-Wait et al., 2009). Participants who met the criteria for high and low social anxiety were invited to the laboratory. Participants with neurological or other severe medical conditions, traumatic brain injuries, current or past psychiatric hospitalization, a current use of psychoactive medication as well as pregnant women and persons under 18 years of age were excluded from the study.

Subsequently, 46 individuals were classified as low (*n* = 34, 73.9% females; age: *M* = 23.50, *SD* = 3.40) and 41 as high socially anxious (*n* = 27, 65.9% females; age: *M* = 21.10, *SD* = 2.76)^2^. To verify the assignment to the high and low socially anxious groups at the time of the laboratory study, we used the German version of the *Social Phobia Inventory* (SPIN; Consbruch et al., 2016) with 17 items to be rated (0 = *not at all* to 4 = *absolutely*). The SPIN is a reliable and valid method to differentiate efficiently between people with and without social phobia (Connor et al., 2000). In our sample SPIN, mean scores of the low (*M* = 16.24, *SD* = 9.26) and high socially anxious group (*M* = 33.54, *SD* = 17.84) are similar to the mean scores of a healthy sample and one with diagnosed social phobia, respectively (Connor et al., 2000).

For pragmatic reasons, most importantly, the availability of 87 participants, we collected our data for the study in two waves. To avoid word of mouth among the students, in the first wave we assessed 40 high and low anxious participants without a social stressor. In the second wave, we invited a further 47 participants into our laboratory and induced stress. Only 25 of the low socially anxious and 22 of the high socially anxious individuals underwent a stress induction procedure whereas the remaining 41 participants did not. Due to the staggered recruitment, we conducted analyses on control variables and found no differences between the stress and no-stress condition (see Table 1). To account for gender differences, we counterbalanced males and females in the high and low anxious groups as well as in the stress and non-stress conditions. See further demographic and questionnaire data in Table 1.

**TABLE 1:** Demographics and questionnaire data.

	No stress (*N* = 40)				Stress (*N* = 47)			
	High socially anxious	Low socially anxious	*t*/χ^2^	*p*	High socially anxious	Low socially anxious	*t*/χ^2^	*p*
*N*	19	21			22	25		
Age	21.95 (*1.39*)	23.43 (*3.28*)	−1.89^*a*^	= 0.069	20.36 (*3.40*)	23.56 (*3.57*)	−3.13^*a*^	= 0.003
	22.73 (*2.64*)				22.06 (*3.81*)		−0.95^*a*^	= 0.345
Gender (female)	10 (*52.6%*)	18 (*85.7%*)	5.20^*b*^	= 0.023^4^	17 (77.3%)	16 (*64%*)	0.99^*b*^	= 0.321
	28 (*70.0%*)				33 (*70.2%*)		<0.01^*b*^	= 0.983
BDI-II	10.37 (*8.26*)	4.52 (*3.87*)	2.82^*a*^	= 0.009	10.50 (*6.94*)	3.64 (*3.26*)	4.24^*a*^	<0.001
	7.30 (*6.92*)				6.85 (*6.29*)		−0.32^*a*^	= 0.752
STAI-T	42.63 (*9.62*)	34.05 (*5.70*)	3.39^*a*^	= 0.002	46.55 (*10.48*)	31.92 (*7.46*)	5.56^*a*^	<0.001
	38.13 (*8.84*)				38.77 (*11.56*)		0.29^*a*^	= 0.775
SPIN	28.84 (*11.01*)	9.48 (*5.76*)	6.87^*a*^	<0.001	27.95 (*11.14*)	6.21 (*4.34*)	3.23^*a*^	= 0.003
	18.68 (*12.99*)				15.81 (*13.52*)		−0.98^*a*^	= 0.329
SSS-V	18.63 (*3.99*)	18.67 (*6.34*)	−0.021^*a*^	= 0.983	16.70 (*5.43*)	24.04 (*5.47*)	−3.05^*a*^	= 0.006
	18.65 (*5.28*)				20.78 (*6.53*)		1.64^*a*^	= 0.105
QMI	2.43 (*0.65*)	2.25 (*0.50*)	0.97^*a*^	= 0.336	2.76 (*0.94*)	2.56 (*1.17*)	0.64^*a*^	= 0.524
	2.33 (*0.58*)				2.65 (*1.06*)		1.71^*a*^	= 0.091

*Means and standard deviations displayed separately for high and low socially anxious individuals. n = Number of participants; BDI = Beck Depression Inventory (Hautzinger et al., 2006); STAI-T = State and trait version of the State-Trait Anxiety Inventory (Laux et al., 1981); SPIN = Social Phobia Inventory (Consbruch et al., 2016); SSS-V = Sensations-Seeking-Scale (Beauducel et al., 2003); QMI = Questionnaire Upon Mental Imagery (Sack et al., 2005). ^a^t score for group comparison. ^b^χ^2^ score for gender ratio comparison.*

The ethics committee approved the procedure. None of the screened participants met our exclusion criteria, they were consequently all invited to the laboratory. After providing informed consent, all participants filled in a questionnaire battery.

### Questionnaires

To ascertain whether our stress induction worked and to account for high levels of dispositional anxiety, we measured state and trait anxiety with the *State-Trait Anxiety Inventory* (STAI; German version: Laux et al., 1981). Depressive symptoms were assessed with the *Beck’s Depression Inventory* (BDI-II; Hautzinger et al., 2006) due to their effects on reward and punishment processing (e.g., Eshel and Roiser, 2010). We administered the *Impulsive Behavior Scale* (UPPS; German version: Schmidt et al., 2008) and *Sensations-Seeking-Scale* (SSS-V; German version: Beauducel et al., 2003) because both values are related to behavior in the *BART* (e.g., Bornovalova et al., 2009). Due to the issue of statistical power, we did not consider the UPPS in our analyses because sensation-seeking and impulsivity have overlapping features and show high correlations with each other (Zuckerman, 1994; Steinberg et al., 2008; Meule et al., 2011).

To examine differences in specific risk estimations between low and high socially anxious individuals, we adapted items of two previously established risk estimation questionnaires, the *Risk of Encounter Questionnaire* and the *Risk of Negative Outcomes Questionnaire* (RNOQ; Hengen and Alpers, 2019). We created items specific to social anxiety for the *Encounter Domain* (e.g., to give a talk) and for the *Outcome Domain* of such encounters (e.g., being laughed at when one gives a talk).

### Stimuli

For the fear-relevant stimuli in the *eBART*, we selected 12 pictures of angry (6 female) and 8 pictures of calm (4 female) facial expressions from the well-validated *NimStim* set (Tottenham et al., 2009). We selected only the most validated facial expressions (Adolph and Alpers, 2010). Calm facial expressions are faces which are perceptually similar to neutral, however, actors are instructed to leave their face more relaxed (Tottenham et al., 2009). We chose these facial expressions as our neutral stimuli because research on face perception has shown that neutral faces are not always perceived as neutral (Donegan et al., 2003; Iidaka et al., 2005). Socially anxious individuals in particular tend to interpret neutral or ambiguous stimuli as negative (Winton et al., 1995; Amin et al., 1998; Amir et al., 2005). To control for head size and form of the facial expressions, we cropped the pictures to an oval form. To standardize the color intensity, we removed the color from the faces and replaced them with shades of gray.

Immediately before and after the *eBART*, participants rated the pictures on a 10-point Likert scale on the dimensions valence (“1 = very unpleasant” to “10 = very pleasant”), arousal (“1 = not at all arousing” to “10 = very arousing”), and, in addition, intensity (“1 = not at all intense” to “10 = very intense”). We added the intensity dimension because anxiety is known to modulate the perception of the intensity and, consequently, the recognition of facial expressions (Kavcioglu et al., 2019).

### Experimental Procedure

At the beginning, in order to comply with standards of informed consent, all participants received a short and unspecific information that they may possibly be asked to give a speech which would be video-taped after the experiment and following rated by professional raters. However, only the 47 participants in the stress condition received the detailed instructions for the *public speaking task* after filling in the questionnaires (Steele and Josephs, 1988; Wieser et al., 2010). They were told that they would need to give a speech at the end of the experiment and that it would be videotaped and later evaluated by experts of the department. They were also informed that the talk would be on a controversial topic and that they would have no time to prepare. Afterwards, they again reported their arousal on the STAI-State. The no-stress condition group did not receive such instructions but also filled in the STAI-State a second time.

Participants then rated the fear-relevant angry and neutral/slightly positive facial expressions used in the *eBART*. Following this, they performed the *eBART* and rated the facial expressions again. They were also asked to rank their motivation to win money and to avoid the fear-relevant stimuli (“0% = not motivated” to “100% = highly motivated”) in the *eBART*. After that, they rated their situational anxiety on the STAI-State and answered questions about their explicit knowledge of the contingencies in the computer task. Finally, they were asked about the plausibility of the stress induction. They were then debriefed and received either a certain amount of money which they won during the task or course credit for their participation^3^.

### Balloon Task With Social Stimuli

The *eBART* is a modified version of the original *BART* (Lejuez et al., 2002) which we previously introduced with spider and butterfly pictures as emotional stimuli (Hengen and Alpers, accepted pending revision). As in the original *BART*, participants were asked to inflate a computer-simulated balloon by pressing a key on the keyboard. With each pump, the balloon grows larger and participants earn a certain amount of virtual money (5 cents). After each pump, participants are free to choose if they want to collect the money earned in this trial (i.e., per balloon) or if they want to continue inflating the balloon. Simultaneously, with each successive pump the risk increases that the balloon explodes. Such explosions concurred with the loss of money earned during this trial.

We set the explosion probability to 1/128 for the first pump. In case the balloon did not explode this probability was increased on the successive pumps to 1/(128 − *n*). This algorithm resulted in an average explosion point after 64 pumps. Thus, a normative and most adaptive point to stop inflating and collect the money earned so far would have been 63 pumps.

Three differently colors of the balloons predicted the contingency of a fear-relevant stimulus after an explosion. The first color indicated a 100% contingency of an angry facial expression, the second a 50% contingency of an angry or a calm facial expression, and the third a 0% probability of an angry facial expression (but a 100% contingency of a calm facial expression). Thus, on successive pumps participants increased the risk of losing the money earned so far. In addition, in the 100 and 50% condition (indicated by balloon color) the risk of an angry facial expression appearing increased. There were 15 balloons of each color/contingency. Thus, the total number of balloons to inflate during the task was 45. For each color, the maximum break point was set to 128. Across all colors, the probability of a balloon exploding was held constant [i.e., 1/(128 − *n*)]. After 6 s, a small square appeared in the middle of the picture and participants had to perform a mouse-click to continue with the next balloon. This procedure ensured that high socially anxious individuals could not visually avoid the aversive stimuli.

**FIGURE 1:**
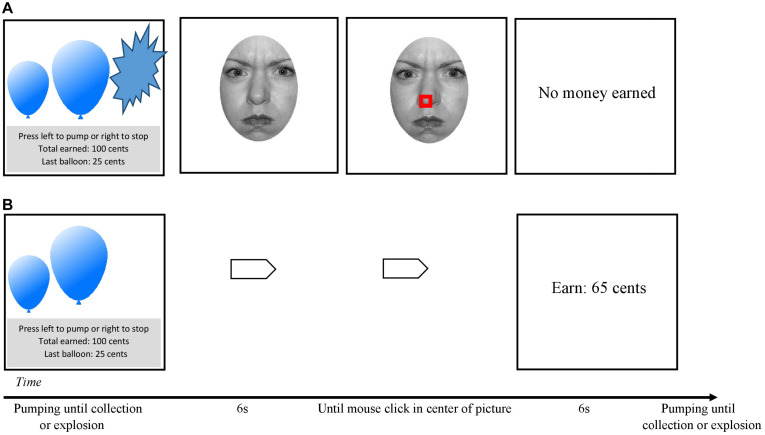
Example of a sequence of the social *eBART* in the 100% contingency condition (all losses accompanied with angry facial expressions). **(A)** Depicts a trial with an explosion: a screen with a balloon was presented. The participants were asked to inflate it. The sequence ends when the participants decided to collect the earned money or the balloon explodes. After an explosion a picture of an angry facial expression was presented. To proceed, participants had to click in the middle of the facial expression (indicated by a little square; Feedback presentation followed and the next trail began. **(B)** Depicts a trial without an explosion. When participants decided to stop inflating before the explosion, the participant only received feedback on their earned money in this trial and the next trial started. The right to publish the actor’s photograph was granted by the authors of the NimStim inventory (Tottenham et al., 2009).

At the beginning, participants were explicitly told which color indicated which contingency, but they had to learn the explosion probability by experience. This procedure increases ecological validity and creates a continuous shift from decisions under uncertainty (probability is yet unknown) to decisions under risk (probabilities become transparent with experience). To avoid confounding effects, the color assigned to each contingency was counterbalanced across participants. Further, contingency blocks were assigned in a randomized manner across the *eBART*. Figure 1 shows an example trial in the 100% contingency condition.

### Statistical Analyses

As a manipulation check for the classification as high and low socially anxious, we compared mean scores of the SPIN between the high and low socially anxious groups with a *t*-test for independent samples. As a manipulation check for the stress induction method, we compared self-reported arousal with the STAI-state scores. Therefore, we conducted a 2 × 2 × 2 mixed ANOVA with the between-subject factors *Social Anxiety* groups (high vs. low) and *Stress* condition (yes vs. no) as well as the within-subject factors *Time* (before stress induction vs. after stress induction).

To check for the emotional relevance of the stimulus material, we ran several 2 × 2 mixed ANOVAs with the between subject factor *Social Anxiety* and the within subject factor *Expression* (angry vs. calm) for each rating dimension (valence, arousal and intensity).

We used three dependent variables as indicators of risk avoidance tendencies. We operationalized risk avoidance behavior by following the recommended procedures for the original BART and computed the average adjusted number of pumps. This is a measure for the number of pumps on trials in which the balloons did not explode. It is more reliable than the total number of average pumps across all balloons and accounts for more between-group variability (Lejuez et al., 2002). As an index for dysfunctional risk avoidance, we used the amount of money earned in the task. In addition, we recorded response times for the decisions to inflate the balloon across all balloon trials as a measure of the cognitive resources invested in the approach-avodiance situation. We calculated the average response time per pump for each participant.

For avoidance behavior across the *eBART*, we grouped the task in three sequential blocks [Block 1 (balloons 1–15), Block 2 (balloons 16–30), Block 3 (balloons 31–45)]. For each block, we calculated the average number of adjusted pumps, the money earned as well as the response times. We then conducted 2 × 2 × 3 mixed ANOVAs with the between subject factor *Social Anxiety* and *Stress* and the within subject factor *Block*.

Furthermore, we used the 100, 50, and 0% contingency trials to manipulate fear-driven avoidance tendencies. For the average adjusted number of pumps, the money earned and the response times, we also ran several 2 × 2 × 3 ANOVAs with the same between subject factors *Social Anxiety* and *Stress* and the within subject factor *Contingency* (100% vs. 50% vs. 0% probability of a fear-relevant stimulus after an explosion).

In order to test our *a priori* hypotheses on the effects of stress, we split the overall 2 × 2 × 3 ANOVA into two separate analyses for each, the stress and the no-stress condition. This resulted in two 2 × 3 ANOVAs with the between subject factor *Stress* and *Social Anxiety* and the relevant within subject factors (*Contingency* and *Block*). We followed this hypothesis-driven rationale even when there was no significant three-way interaction involving *Stress* in the overall test. However, in every instance when the overall test did not support it, we marked *a priori* contrasts as exploratory.

In addition, we examined the distinct components of risk estimates by using two 2 × 2 ANOVAs with *Stress* and *Social Anxiety* as between subject factors and either risk estimates of fear-relevant encounters or risk estimates of negative outcomes of such encounters as dependent variables. In case of significant main effects and interactions, these were further specified with post-hoc comparisons. In case of violated sphericity, Greenhouse-Geisser’s we adjusted degrees of freedom appropriately.

## Results

### Manipulation Check for the Stress Induction

**FIGURE 2:**
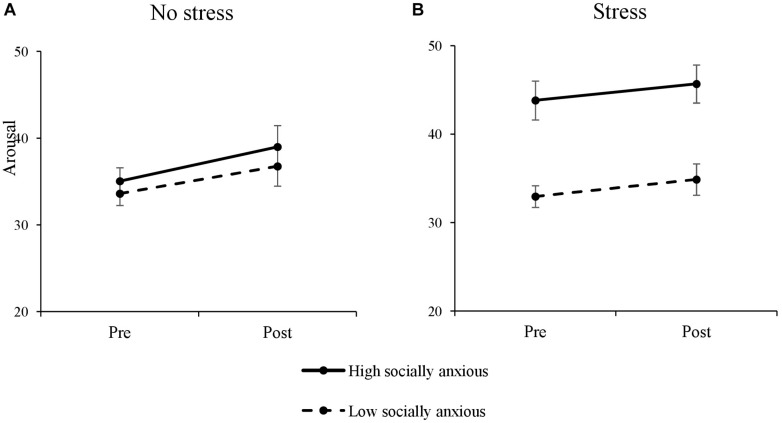
Means of the sums scores of the STAI-S before and after the stress induction. **(A)** The group with no stress. **(B)** The group with the social stress induction. Error bars reflect the standard error of the mean.

We tested the effectiveness of our stress induction by assessing situational arousal with the STAI- state before and after the stress induction; see Figure 2. Overall, participants experienced more arousal after the stress induction, indicated by a significant main effect of *Time*, *F*(1, 83) = 9.44, *p* = 0.003, $\eta_{p}^{2}$ = 0.10; *t*(86) = 3.04, *p* = 0.003. Furthermore, high socially anxious individuals were more aroused at both times than low socially anxious individuals; main effect of *Social Anxiety*, *F*(1, 83) = 14.02, *p* < 0.001, $\eta_{p}^{2}$ = 0.15; Pre: *t*(65.76) = 3.65, *p* = 0.001, Post: *t*(80.42) = 3.12, *p* = 0.003. In addition, the effectiveness of our stress induction varied with the level of social anxiety; interaction *Stress* × *Social Anxiety*, *F*(1, 83) = 7.07, *p* = 0.009, $\eta_{p}^{2}$ = 0.08. Thus, we conducted separate 2 × 3 ANOVAs for each stress condition.

Without the stress induction, there were no differences between the anxiety groups, *F*(1, 38) = 0.61, *p* = 0.439, $\eta_{p}^{2}$ = 0.02; Pre: *t*(38) = 0.70, *p* = 0.491, Post: *t*(38) = 0.67, *p* = 0.508. However, under stress, high socially anxious individuals generally reported more arousal than low socially anxious individuals on both time points, indicated by a significant main effect of *Social anxiety*, *F*(1, 45) = 20.45, *p* ≤ 0.001, $\eta_{p}^{2}$ = 0.3, Pre: *t*(33.42) = 4.30, *p* < 0.001, and Post: *t*(45) = 3.91, *p* < 0.001. None of the other main effects and interactions were significant, all *F*s ≤ 3.64, all *p*s ≥ 0.060, all $\eta_{p}^{2}$s ≤ 0.04.

To further check, if only high socially anxious individuals were assigned to the stress condition we conducted an univariate ANOVA with the between-subject factors *Stress* and *Social Anxiety* and the dependent variable STAI-S. Our results showed a significant main effect of both, *Stress*, *F*(1, 83) = 6.17, *p* = 0.015, $\eta_{p}^{2}$ = 0.07, and *Social Anxiety*, *F*(1, 83) = 14.19, *p* < 0.001, $\eta_{p}^{2}$ = 0.15, as well. In addition, there was also a significant interaction of both factors, *F*(1, 83) = 8.34, *p* = 0.005, $\eta_{p}^{2}$ = 0.09, that might indicated that especially high socially anxious with elevated baseline levels of state anxiety, were assigned to stress condition.

To sum up, high socially anxious individuals in the stress condition showed already elevated stress levels at the very beginning of the task.

### Manipulation Check for the Stimulus Material

To determine the emotional relevance of the stimulus material, we conducted separate ANOVAs for each rating dimension. For valence, all participants rated angry facial expressions as more negative than calm expressions, indicated by a significant main effect of *Expression*, *F*(1, 85) = 260.47, *p* < 0.001, $\eta_{p}^{2}$ = 0.75. There was no main effect of *Social Anxiety*, *F*(1, 85) = 2.61, *p* = 0.110, $\eta_{p}^{2}$ = 0.03, and no significant interaction, *F*(1, 85) = 0.49, *p* = 0.486, $\eta_{p}^{2}$ = 0.01.

For arousal, all participants perceived angry facial expressions as more arousing than calm ones, main effects of *Expression*, *F*(1, 85) = 201.10, *p* < 0.001, $\eta_{p}^{2}$ = 0.04. In addition, high compared to low socially anxious individuals rated angry faces as more arousing, indicated by the significant main effect of *Social Anxiety*, *F*(1, 85) = 5.78, *p* = 0.018, $\eta_{p}^{2}$ = 0.06, *t*(86) = 2.44, *p* = 0.017, but there were no differences for calm faces, *t*(86) = 1.86, *p* = 0.066. There was no significant interaction, *F*(1, 85) = 2.09, *p* = 0.152, $\eta_{p}^{2}$ = 0.02.

For intensity, angry faces were perceived as more intense than calm faces by all individuals; main effect of *Expression*, *F*(1, 85) = 610.53; *p* < 0.001, $\eta_{p}^{2}$ = 0.88. However, high compared to low socially anxious individuals perceived angry faces, *t*(85) = 1.36, *p* = 0.177, and calm faces, *t*(85) = 1.84, *p* = 0.070, as more intense, which was indicated by the significant main effect of *Social Anxiety*, *F*(1, 85) = 4.01, *p* = 0.048, $\eta_{p}^{2}$ = 0.05. There was no significant interaction, *F*(1, 85) = 0.22, *p* = 0.638, $\eta_{p}^{2}$ = 0.00. Figures to illustrate the ratings are provided in the Supplementary Material.

To conclude, our stimulus material proved to be emotionally relevant and, especially for high socially anxious individuals, arousing.

### Effects of Social Anxiety and Stress on Decision-Making

#### Risk Avoidance of Emotionally Relevant Stimuli

**FIGURE 3:**
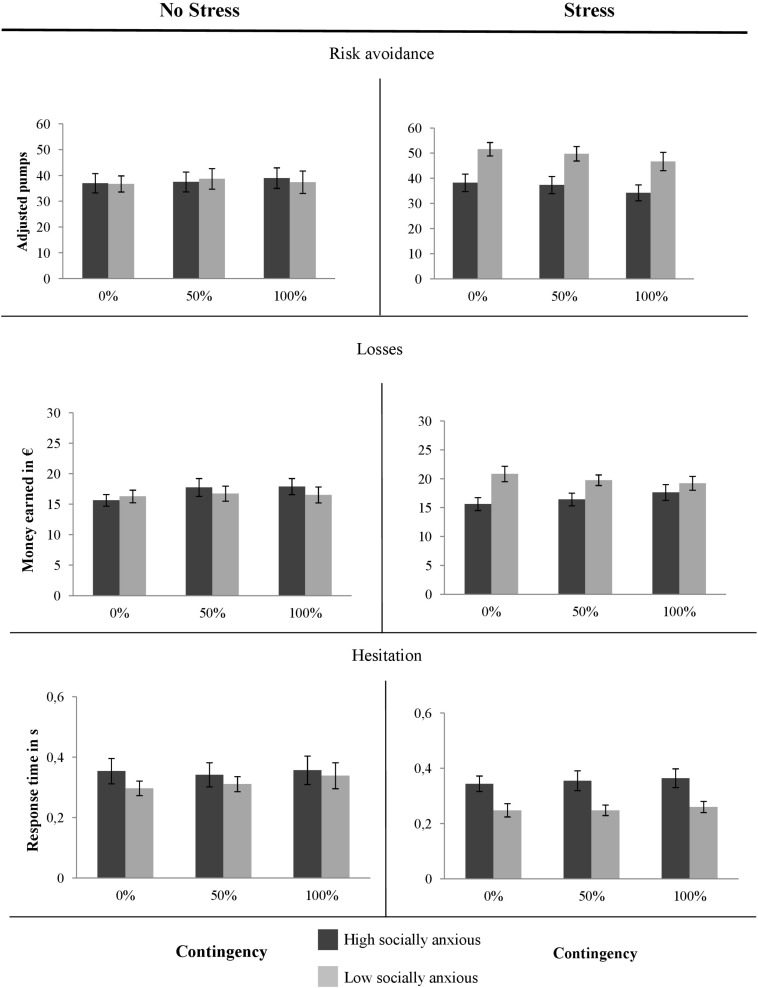
Means of the adjusted number of pumps, of the earned money and the time per pump depending on the contingency of a fear-relevant stimulus. Error bars reflect the standard error of means.

The results of the dependent variables for the different contingencies (0% vs. 50% vs. 100%) are presented in Figure 3. To test the assumption that stress affects high and low socially anxious individuals differently, we conducted a 2 × 2 × 3 ANOVA for the averaged adjusted pumps. There was a significant interaction between *Social Anxiety* and *Stress*, *F*(1, 83) = 3.94, *p* = 0.050, $\eta_{p}^{2}$ = 0.05, and between *Contingency* and *Stress*, *F*(2, 83) = 3.17, *p* = 0.045, $\eta_{p}^{2}$ = 0.04. No main effects and three-way interactions were observed, all *F*s ≤ 3.68, all *p*s ≥ 0.058, all $\eta_{p}^{2}$s ≤ 0.04.

##### Exploratory analysis of emotional risk avoidance

Without stress, there were no effects for *Social Anxiety, F*(1, 38) = 0.00, *p* = 0.967, $\eta_{p}^{2}$ = 0.00, nor for *Contingency*, *F*(2, 76) = 0.46, *p* = 0.631, $\eta_{p}^{2}$ = 0.01, nor an interaction between the two factors, *F*(2, 76) = 0.41, *p* = 0.655, $\eta_{p}^{2}$ = 0.01. Thus, without stress, high socially anxious individuals behaved in the same way as low socially anxious individuals.

Under stress, however, we observed a significant main effect of *Contingency*, *F*(2, 90) = 3.17, *p* = 0.045, $\eta_{p}^{2}$ = 0.04, but no significant interaction with *Social Anxiety*, *F*(2, 90) = 0. 50, *p* = 0.951, $\eta_{p}^{2}$ = 0.00. On an individual level, all participants more frequently avoided trials with a 100% contingency than trials with a 50% contingency, *t*(46) = −2.03, *p* = 0.049, and trials with a 100% contingency of fear-relevant stimuli compared to ones with 0% contingency, *t*(46) = −2.47, *p* = 0.017. However, there were no differences in the number of pumps between the 50 and 0% contingency trials, *t*(46) = 0.87, *p* = 0.3.88.

The significant main effect of *Social Anxiety*, *F*(1, 45) = 9.57, *p* = 0.003, $\eta_{p}^{2}$ = 0.18, indicated that high socially anxious individuals had an overall risk avoidance tendency, regardless of the contingency between the decision and fear-relevant outcomes. We conducted further post-hoc tests to account for differences within high and low socially individuals between the stress and no-stress conditions. In all contingency conditions, high socially anxious individuals under stress were equally averse to risk as high socially anxious individuals without stress, all *t*s ≤ 0.95, all *p*s ≥ 0.351. Interestingly, low socially anxious individuals under stress increased their risk-taking in the stress compared to the no-stress condition, 0% contingency: *t*(44) = 3.64, *p* ≤ 0.001, 50% contingency: *t*(44) = 2.30, *p* = 0.026. However, low socially anxious individuals did not differ in the 100% contingency between the stress and no-stress condition, *t*(44) = 1.65, *p* = 0.105.

To sum up, stress affected high and low socially anxious individuals differently: whereas high socially anxious individuals remained risk-averse independently of the stress conditions, low socially anxious individuals became more willing to take risks, especially when the risk of an emotional stimulus was rather low.

#### Monetary Losses Due to Risk Avoidance

To account for dysfunctional avoidance behavior operationalized by the money earned, we again conducted the overall 2 × 2 × 3 ANOVA and found a significant interaction between *Social Anxiety* and *Stress*, *F*(1, 83) = 5.49, *p* = 0.021, $\eta_{p}^{2}$ = 0.06, but no other significant effects, all *F*s ≤ 2.92, all *p*s ≥ 0.091, all $\eta_{p}^{2}$s ≤ 0.03.

##### Exploratory analysis of monetary losses

Without stress, high and low socially anxious individuals did not differ, all *F*s ≤ 3.68, all *p*s ≥ 0.058, all $\eta_{p}^{2}$s ≤ 0.04. In contrast, under stress, high socially anxious individuals earned less money, *Social Anxiety F*(1, 45) = 10.11, *p* = 0.003, $\eta_{p}^{2}$ = 0.18. This was not affected by the contingency of fear-relevant stimuli; all other effects did not reach significance, all *F*s ≤ 3.68, all *p*s ≥ 0.058, all $\eta_{p}^{2}$s ≤ 0.04.

#### Response Times of Decisions

To analyze if stress affects the response time of high and low socially anxious individuals differentially, we again conducted the overall 2 × 2 × 3 ANOVA. There was a significant main effect of *Social Anxiety*, *F*(1, 45) = 5.37, *p* = 0.023, $\eta_{p}^{2}$ = 0.06, but no other significant differences or interactions, all *F*s ≤ 1.93, all *p*s ≥ 0.148, all $\eta_{p}^{2}$s ≤ 0.02.

##### Exploratory analysis of response times

Without a stressor, high and low socially anxious individuals did not differ in their response times, all *F*s ≤ 1.21, all *p*s ≥ 0.298, all $\eta_{p}^{2}$s ≤ 0.03.

However, under stress, high socially anxious individuals had slower response times, main effect of *Social Anxiety*: *F*(1, 45) = 8.59, *p* = 0.005, $\eta_{p}^{2}$ = 0.16, regardless of whether they were at risk of a fear-relevant stimulus; no main effect of *Contingency*, *F*(2, 90) = 0.736, *p* = 0.482, $\eta_{p}^{2}$ = 0.02, no significant interaction, *F*(2, 90) = 0.09, *p* = 0.918, $\eta_{p}^{2}$ = 0.00.

To conclude, high socially anxious individuals showed same response times independent of a social stressor. The social stressor reduced response times only in those with low social anxiety. This finding is in line with the other risk-related dependent variables.

#### Risk Avoidance Across the Task

**FIGURE 4:**
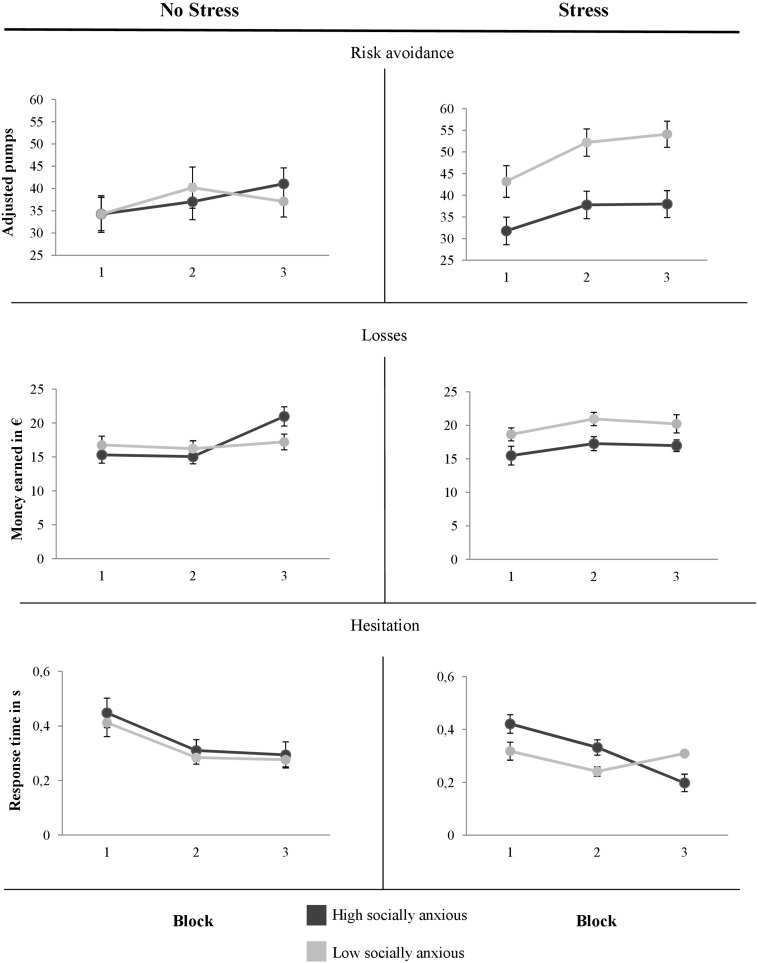
Means of the adjusted number of pumps, of the earned money and the time per pump depending on the block (1 = balloon 1–10; 2 = balloon 11–20; 3 = 2 balloon 1–30) throughout the *eBART.* Error bars reflect the standard error of means.

In this section, we report the results from the 2 × 2 × 3 ANOVAs with the between subject factors *Stress* and *Social Anxiety* and dependent measures as above. Instead of the within subject factor *Contingency*, we added the within subject factor *Block* to account for time effects on the risk-related variables across the task. The overall findings of the dependent variables across the *eBART* are presented in Figure 4.

To find out whether high and low socially anxious individuals systematically differ in their risk avoidance depending on stress, we ran the 2 × 2 × 3 ANOVA for the number of adjusted pumps across the task. We had a significant main effect of *Block*, *F*(2, 166) = 15.83, *p* < 0.001, $\eta_{p}^{2}$ = 0.16, which indicated that all individuals inflated the balloons more when the task proceeded. Furthermore, high socially anxious compared to low socially anxious individuals inflated the balloons to a lesser degree across the *eBART*, *F*(1, 83) = 4.50, *p* = 0.037, $\eta_{p}^{2}$ = 0.05. A significant interaction between *Stress* and *Social Anxiety* showed that stress affected high and low socially anxious individuals differently, *F*(1, 83) = 4.50, *p* = 0.037, $\eta_{p}^{2}$ = 0.08. There were no other significant differences, all *F*s ≤ 2.80, all *p*s ≥ 0.098, all $\eta_{p}^{2}$s ≤ 0.03.

##### Exploratory analysis of risk avoidance across the task

Without stress, high and low socially anxious individuals did not differ in the average number of pumps across the task: significant main effect of *Block*, *F*(1, 38) = 0.00, *p* < 0.983, $\eta_{p}^{2}$ = 0.00. Indeed, they all had the same increase in the adjusted number of pumps, *F*(2, 76) = 3.48, *p* < 0.001, $\eta_{p}^{2}$ = 0.25. Further, all individuals inflated the balloon more in Block 2, *t*(39) = 2.10, *p* = 0.043, and Block 3, *t*(39) = 2.28, *p* = 0.028. There was no difference between Block 2 and 3, *t*(39) = 0.16, *p* = 0.878. The results indicated that the adjusted number of pumps did not differ as a function of *Social Anxiety*, *F*(2, 76) = 1.79, *p* = 0.176, $\eta_{p}^{2}$ = 0.05.

Under stress, all individuals learned to adapt their behavior and inflated the balloons more, indicated by a significant main effect of *Block*, *F*(2, 90) = 14.98, *p* < 0.001, $\eta_{p}^{2}$ = 0.25. This difference was due to a larger increment of pumps from Block 1 to Block 2, *t*(46) = 4.56, *p* < 0.001, and from Block 1 to Block 3, *t*(46) = 4.60, *p* < 0.001. However, there was no difference between Block 2 and 3, *t*(46) = 0.74, *p* = 0.463. Further, the significant main effect of *Social Anxiety* revealed that high socially anxious individuals inflated the balloon less across the entire task, *F*(1, 45) = 11.38, *p* = 0.002, $\eta_{p}^{2}$ = 0.20. In addition, there was no significant interaction between *Social Anxiety* and *Block*, *F*(2, 90) = 0.98, *p* = 378, $\eta_{p}^{2}$ = 0.02.

To summarize, learning might have taken place in the first trials of the *eBART*. Furthermore, when being stressed, low socially anxious individuals inflated the balloon more and learned to adapt their behavior over the course of the task. High socially anxious participants remained risk-averse regardless of whether they were exposed to stress or not.

#### Losses Due to Risk Avoidance

We analyzed differences between high and low socially anxious individuals in the amount of money that they earned across the task. The 2 × 2 × 3 ANOVA showed a significant main effect of *Block*, *F*(2, 166) = 5.45, *p* = 0.005, $\eta_{p}^{2}$ = 0.06, as well as a significant interaction between *Block* and *Stress*, *F*(2, 166) = 4.10, *p* = 0.018, $\eta_{p}^{2}$ = 0.05, and *Social Anxiety* and *Stress*, *F*(1, 83) = 4.77, *p* = 0.032, $\eta_{p}^{2}$ = 0.05. The other main effects and interactions did not reach significance, all *F*s ≤ 2.35, all *p*s ≥ 0.099, all $\eta_{p}^{2}$s ≤ 0.03.

##### Exploratory analyses of losses across the task

Without the social stressor, all individuals earned more money as the task proceeded, *F*(2, 76) = 8.47, *p* < 0.001, $\eta_{p}^{2}$ = 0.18. Furthermore, without stress, groups did not differ in how much money they earned, *F*(1, 38) = 0.07, *p* = 0.792, $\eta_{p}^{2}$ = 0.00. However, the increase in the money earned across the task varied as a function of *Social anxiety*, *F*(2, 76) = 5.13, *p* = 0.009, $\eta_{p}^{2}$ = 0.12.

To account for this interaction effect, we conducted a separate one-way ANOVA for high and low socially anxious individuals each. High socially anxious individuals learned to inflate the balloon more and increased their money earned as the task proceeded, *F*(2, 36) = 12.85, *p* < 0.001, $\eta_{p}^{2}$ = 0.42. This main effect was driven by a significant increase in money earned from Block 2 to 3, *t*(18) = 4.97, *p* = 0.043, but not from Block 1 to 2, *t*(18) = 0.19, *p* = 0.850. Interestingly, low socially anxious individuals did not earn more money across the task, *F*(2, 40) = 0.31, *p* = 0.734, $\eta_{p}^{2}$ = 0.02.

When they were stressed, high socially anxious individuals compared to low socially anxious individuals also earned less money across the task, which was indicated by the significant main effect of *Social anxiety*, *F*(1, 45) = 10.11, *p* = 0.003, $\eta_{p}^{2}$ = 0.18. There were no other significant differences, all *F*s ≤ 2.12, all *p*s ≥ 0.127, all $\eta_{p}^{2}$s ≤ 0.05.

To sum up, low socially anxious individuals took more risks under stress and earned more money across the *eBART*. High socially anxious individuals remained risk-averse and therefore earned less.

#### Decision Response Time

For the response time in individual decisions, the overall 2 × 2 × 3 ANOVA showed that all individuals regardless of their degree of social anxiety and being stressed inflated the balloon faster as the task proceeded, indicated by the significant main effect of *Block*, *F*(2, 162) = 45.87, *p* < 0.001, $\eta_{p}^{2}$ = 0.36. Furthermore, regardless of whether they were stressed, high socially anxious individuals took longer in their decisions to inflate the balloons across the entire task, *F*(1, 81) = 4.57, *p* = 0.036, $\eta_{p}^{2}$ = 0.05. There were no other significant effects, all *F*s ≤ 2.12, all *p*s ≥ 0.127, all $\eta_{p}^{2}$s ≤ 0.05.

##### Exploratory analysis of response times across the task

Without stress, all individuals reduced response times across trials in the task, *F*(1.21, 43.45) = 19.89, *p* < 0.001, $\eta_{p}^{2}$ = 0.36. This effect was due to early faster response times from Block 1 to 2, *t*(37) = 4.58, *p* < 0.001, but not from Block 2 to 3, *t*(37) = 1.07, *p* = 0.292. High and low socially anxious individuals did not differ in their response times while inflating the balloons during the *eBART*, *F*(1, 36) = 0.28, *p* = 0.603, $\eta_{p}^{2}$ = 0.01.

Under stress, all individuals inflated the balloons faster as the task proceeded, *F*(1.56, 70.19) = 27.31, *p* < 0.001, $\eta_{p}^{2}$ = 0.38. Their response time decreased from Block 1 to 2, *t*(46) = 2.94, *p* = 0.005, and from Block 2 to 3, *t*(4) = 4.97, *p* < 0.001. However, high socially anxious individuals responded more slowly across the whole task, *F*(1, 45) = 8.57, *p* = 0.005, $\eta_{p}^{2}$ = 0.16. Again, this effect was a result of faster responses in low socially anxious individuals. Furthermore, the response time did not differ as a function of social anxiety, *F*(1, 45) = 8.57, *p* = 0.005, $\eta_{p}^{2}$ = 0.16. Under stress, high socially anxious individuals responded as slowly as under no stress, whereas individuals low in social anxiety sped up their responses.

### Risk Estimations: Questionnaire Scores

For the risk questionnaires, we conducted two 2 × 2 univariate ANOVAs with the factors *Stress* and *Social anxiety* and the risk estimates of socially relevant encounters and negative outcomes as dependent variables.

For risk estimates of socially relevant encounters, stress affected high and low socially anxious individuals equally: individuals in the stress condition gave lower risk estimates of socially relevant encounters than individuals in the no-stress condition, *F*(1,83) = 4.79, *p* = 0.031, $\eta_{p}^{2}$ = 0.06. Counterintuitively, high socially anxious compared to low socially anxious individuals gave lower risk estimates of such encounters, *F*(1,83) = 4.94, *p* = 0.029, $\eta_{p}^{2}$ = 0.06. There was no significant interaction between *Stress* and *Social anxiety*, *F*(1,83) = 0.33, *p* = 0.566, $\eta_{p}^{2}$ ≤ 0.00.

For risk estimates of negative outcomes of socially relevant encounters, there was only a significant main effect of *Social anxiety*, *F*(1,83) = 19.26, *p* ≤ 0.001, $\eta_{p}^{2}$ = 0.19, in the way that high socially anxious rated the risk of negative outcomes of socially relevant encounters higher than low socially individuals. There were no other significant results, all *F*s ≤ 3.78, all *p*s ≥ 0.055, all $\eta_{p}^{2}$s ≤ 0.04.

To conclude, both stress and anxiety led to lower risk estimates of socially relevant encounters. Interestingly, the estimated risk of negative outcomes of such encounters was only affected by social anxiety in the way that high socially anxious rated the risk of negative outcomes higher than low socially anxious individuals.

## Discussion

Stress and anxiety can both affect human information processing (Ekman and Davidson, 1994; Lorian and Grisham, 2010; Starcke and Brand, 2016). In the present study, we systematically investigated the distinct effects of stress and anxiety on risk-taking behavior. We examined risk-taking in an adapted version of a well-established risk-taking paradigm for high and low socially anxious individuals, the social *eBART*. To induce stress, participants were led to anticipate a socially evaluative task.

The main results confirmed that stress made the difference: It induces low socially anxious individuals to take more risks – independently of socially relevant stimuli – and therefore earn more money. Without stress, there was no influence of either of the risk-taking parameters. Interestingly, stress and anxiety had opposite effects on the perceived risk of socially relevant events. On the one hand, stress resulted in higher risk estimates in socially relevant encounters. On the other hand, anxiety triggered lower risk estimates of such encounters. Whereas anxiety affected risk estimates of negative outcomes of socially relevant encounters, stress did not.

This is the second study that used the *eBART* as a measure for risk avoidance in anxiety when competing approach-avoidance tendencies are present (Hengen and Alpers, accepted pending revision). Our findings show that the type of fear might moderate the extent of avoidance in the task. In our first study with the *eBART*, spider-fearful individuals overall showed heightened risk avoidance in the *eBART*, even in the absence of fear-relevant stimuli. In the present study, this was not the case for high socially anxious individuals. When they were confronted with socially fear-relevant material in the *eBART*, they did not differ from low socially anxious participants. This is in line with previous research that shows findings with fear-relevant spider pictures cannot be generalized directly to facial expressions (Alpers et al., 2011; Diemer et al., 2015; Berdica et al., 2018). This may be due to the cognitive complexity of social anxiety compared to specific phobias. Facial expressions are of emotional relevance to all individuals and are processed preferentially in low anxious individuals as well as in high anxious ones (Alpers and Gerdes, 2007; Kavcioglu et al., 2019). Previous research highlighted the importance of the social context when using facial expressions for anxious individuals (Richards et al., 2007; Wieser and Brosch, 2012; Bublatzky and Alpers, 2017; Bublatzky et al., 2017).

In addition, our finding of more risk avoidance in high socially anxious individuals corresponds with self-reported risk aversion in other recent work (Stamatis et al., 2020). However, the same study reported a small but significant correlation between social anxiety and incentivized gambling attractiveness (especially so in a genetic risk group), which they interpret as a specific component of behavioral risk-taking (Stamatis et al., 2020). Although their task to assess gambling attractiveness addresses decision-making under risk as well, their task also differs from ours. Interestingly, Stamatis et al. (2020) found that high socially anxious individuals who were better able to estimate reward probabilities in the experiment took more risks. Because reward probabilities in the *BART* are not easily assessable (Lejuez et al., 2002; Hunt et al., 2005) this may contribute to the differences between their and our findings; only more research can resolve this issue.

In order to interpret the group differences, it is important to more closely consider characteristics of our low socially anxious control group. Because their mean scores on social anxiety were comparable to a representative German sample of healthy individuals (e.g., Sosic et al., 2008) we have no reason to assume that this group behaved differently from the norm. Importantly, their behavior is in line with previous research that indicates heightened risk-taking in non-anxious participants when being stressed (Porcelli and Delgado, 2009; Putman et al., 2010; Starcke and Brand, 2016). Thus, we consider the change in behavior that we observed in the low socially anxious group as a benchmark for the comparisons with the high socially anxious participants who clearly score higher in social anxiety than the normative sample (Sosic et al., 2008). Because we have presented theoretically supported hypotheses for highly anxious participants – that they are more avoidant than the norm – we interpret the group differences as a result of their social anxiety.

Different from other decision-making tasks (e.g., *IGT*; Bechara et al., 1994), heightened risk-taking behavior in the *eBART*, as in the original task (Lejuez et al., 2002), is adaptive and riskier decisions are rewarded (up to a certain extent). In addition, the fixed probability schedule gives participants the opportunity to learn from experience and to adapt their behavior to more risk-taking. Interestingly, as our findings indicated, learning in the *eBART* took place in the first third of the task and remained stable. However, stress only affects the learning curve of low socially anxious individuals as they adapted their behavior to the task more quickly and earned more money. This finding is in line with previous studies that show how individuals under stress might focus more on rewards than losses (Petzold et al., 2010). However, when being dispositionally anxious, the adaptive effects of stress in tasks that reward risk-taking is undermined. As in previous studies, anxiety might mitigate the reward sensitivity and consequently result in dysfunctional reward processing (Held-Poschardt et al., 2018).

As stress and anxiety are known to influence information processing, we assessed response times as an indirect measure of decisional conflict in this study. Especially when decisions include emotionally relevant options to choose from, efficient processing of rewards and losses is necessary to adapt one’s behavior to the demands of the task at hand (Botvinick et al., 2001; Larson et al., 2013). In our study, this should have been particularly the case for high socially anxious individuals when they are confronted with fear-relevant stimuli. Interestingly, only stress affected the processing of the conflict between approach and avoidance such that low socially anxious individuals responded faster to inflate the balloon than high socially anxious individuals. However, this finding must be interpreted cautiously, as response time is only an indirect measure of information processing. Contrary to conflict-driven tasks, the *eBART* does not require faster response times as an index of task performance. Thus, response times in this paradigm might not be an adequate representation of deficient conflict processing. Slowed responses also may indicate a stronger approach-avoidance conflict as high socially anxious individuals might have evaluated costs and benefits more intensively.

Previous studies have shown that both acute stress (Starcke and Brand, 2016) and anxiety as a trait (Hengen and Alpers, 2019) affect risk perception. However, the systematic investigation of the distinct aspect of fear-relevant risk estimations has indicated that stress and anxiety have opposite effects. Stress prompts heightened estimates of socially relevant encounters; high anxious individuals merely overestimated the risk of negative outcomes of such encounters. Our findings again emphasize the importance of systematically investigating the distinct components of risk perception, namely emotionally relevant encounters and the negative outcomes of such encounters, and to consider the distinct effects of stress as well as anxiety. Simultaneously, we replicated findings of a recent study (Hengen and Alpers, 2019) and showed that high anxious individuals only gave higher risk estimates of the negative outcomes.

There are some limitations to consider. First, we do not have strong evidence that our stress induction was sufficient to induce heightened arousal levels. We observed that especially high socially anxious individuals in our stress condition had elevated levels of state anxiety at the baseline. Thus, the effects in state anxiety might not have been caused by our stress induction. One reason why the stress effect did not turn out more clearly may have been that all participants in the high socially anxious group were anxious about the stress induction. This was because all individuals received an unspecific information about an upcoming public speech in the study information and informed consent at the very beginning of the study. Thus, this announcement may have diminished the effect of the actual intervention and rendered the three-way interaction between *Stress*, *Time* and *Group* non-significant. Indeed, high socially anxious individuals in our sample report higher state anxiety at both time points. In addition, and more importantly, the STAI-S may not have been most sensitive measurement to specifically assess current arousal (Balsamo et al., 2013). Thus, although the manipulation check was not unambiguous, the pattern of the main outcomes was. The experimental data clearly show differences in form of significant two-way interactions between stress conditions and anxiety groups. Because we have no reason to believe that these effects were driven by anything else but the experimental manipulation, we are convinced that our stress induction was sufficiently effective. However, we recommend more specific measures to assess the effectiveness of the manipulation in future studies.

Third, we need to look at the assignment of the participants to the stress and non-stress conditions. Due to pragmatic considerations concerning recruitment (e.g., word of mouth between participants in the stress and no- stress condition), we first assessed the non-stress condition and then the stress condition. One may thus argue that stress effects might be due to *a priori* differences in the samples. However, as we did check and control for such differences, this does not appear to be a problem. Furthermore, regarding clinical implications, we only assessed individuals with variations of social anxiety and no clinical sample. However, our high anxious sample had values in the same range as patients with a social anxiety disorder (Connor et al., 2000). Consequently, our findings may be generalized to socially phobic individuals. Last, a balloon explosion in the *eBART* resulted in a monetary loss as well as in the appearance of a fear- relevant stimulus. Therefore, more risk-averse behavior under stress might be the result of heightened sensitivity to potential losses (Hartley and Phelps, 2012). However, other findings of support the idea that risk aversion triggers avoidance behavior under uncertain conditions, not the sensitivity to potential losses (Charpentier et al., 2017).

## Conclusion

Although stress and anxiety are important affective states that play a key role in many mental disorders (Ekman and Davidson, 1994), little is known about their distinct effects. This study systematically examined these meaningful constructs in a novel decision-making paradigm that models competing approach-avoidance conflicts. Our findings support the idea that anxiety and stress have interacting effects on behavior. We conclude that anxious individuals do not always evaluate risks in a dysfunctional way. In the context that we examined, their avoidance remained at the same level even when this incurred costs for them; in this they differed from the low anxious control participants.

## Data Availability Statement

The datasets analyzed for this study is stored on MADATA-Mannheim Research Data Repository (doi: 10.7801/340).

## Ethics Statement

The studies involving human participants were reviewed and approved by Ethics Committee of the University of Mannheim. The patients/participants provided their written informed consent to participate in this study. Written informed consent was obtained from the individual(s) for the publication of any potentially identifiable images or data included in this article.

## Author Contributions

KH and GA contributed to the design and implementation of the research, analysis of the results, and writing of the manuscript. Both authors contributed to the article and approved the submitted version.

## Conflict of Interest

The authors declare that the research was conducted in the absence of any commercial or financial relationships that could be construed as a potential conflict of interest.

## Footnotes

1 Referring to previous results of the *eBART* (Hengen and Alpers, accepted pending revision) and to similar approach-avoidance paradigms (e.g., Pittig et al., 2018), effect sizes for the influence of anxiety as well as induced stress were transformed into Cohen’s *f* and set as a large effect of *f* = 0.40. We conducted post-hoc power analyses (GPower; Faul et al., 2009) with a given α = 0.05 and a given sample size of 87 participants. The statistical power for all main effects and interactions was ≥0.96 and thus interpreted as sufficiently for our analyses.

2 Additional analyses for the effects of gender on risk avoidance are described in more detail in the Supplementary Material.

3 31 participants (48.4% high socially anxious and 25.8% under stress) received a performance-based monetary incentive, the other 56 participants (46.4% high socially anxious and 69.6% under stress) were rewarded with course credits. Although performance-based rewarded and credit rewarded participants were not equally distributed across groups, they did not differ in any of the dependent variables, all *t*s ≤ 1.60, all *p*s ≥ 0.114. Thus, the following observed effects were not influenced by the type of compensation.
