# Supplementary material for: Stress Makes the Difference: Social Stress and Social Anxiety in Decision-Making Under Uncertainty
Source: Front Psychol. 2021 Feb 22;12:578293. doi: 10.3389/fpsyg.2021.578293 (PMC7937725; doi:10.3389/fpsyg.2021.578293)
Supplement: Supplementary file 1 [file Data_Sheet_1.docx]

Supplementary Analyses

# Gender and stress interaction

We conducted several 2 x 2 x 3 ANOVAs with the two between subject factors *Gender* (females vs. males) and *Stress* (stress vs. no stress) as well as the within subject factor *Contingency* (for emotional decision-making) or *Block* (for decision-making throughout the task), respectively. We used the average number of adjusted pumps in the *eBART* as the risk-related dependent variable. We did not include *Social Anxiety* as a factor due to interpretation and power issues. Additionally, females and males did not differ in their degree of social anxiety, *t*(81) = 0.07, *p* = 943; neither did the stress- and no stress-groups. (see Table 1).

## Gender and stress effects on emotional decision-making

Overall, females inflated the balloon to a lesser degree than males, *F*(1, 83) = 6.40, *p* < .013, *η²_p_* = .07. Additionally, there was a significant interaction of *Contingency* and *Stress*, *F*(2, 166) = 4.25, *p* < .016, *η²_p_* = .07. This effect was driven by more pumps in the 0% contingency condition in stressed individuals compared to non-stressed ones, *t*(85) = 2.51, *p* = 014. There were no differences between stressed and non-stressed individuals in the 50%, *t*(85) = 1.62, *p* = 0.110, or 100% contingency conditions, *t*(85) = 0.69, *p* = 489. There was no significant interaction between *Stress* and *Gender*, *F*(1, 83) = 1.93, *p* < .169, *η²_p_* = .02, and no other significant main and interaction effects. all *F*s ≤ 1.36, all *p*s ≥ .259, all *η²_p_* s *≤* .02.

## Gender and stress effects in decision-making throughout the task

Overall, all individuals inflated the balloons to a higher degree as the task proceeded, *F*(2, 166) = 13.57, *p* < .001, *η²_p_* = .14. This effect was due to an increased number of pumps from Block 1 to Block 2, *t*(86) = 4.67, *p* < .001, but not from Block 2 to Block 3, *t*(86) = 0.63, *p* = 529. Additionally, females behaved more risk-averse than males throughout the whole task, *F*(1, 83) = 8.03, *p* < .006, *η²_p_* = .09.

# Speed-accuracy-trade-offs between groups and conditions

To investigate speed-accuracy trade-offs we conducted post-hoc correlational analyses. First, we used the whole sample to correlate the response time across the entire task as an indicator of speed, and the total adjusted number of pumps as an indicator of accuracy. In the next step, we examined correlations between the stress conditions and anxiety groups and compared them to each other with a *z*-test for the comparison for correlations.

## Speed-accuracy trade-offs between groups and conditions

Overall, response time correlated negatively with task performance, *r* = -.41, *p* ≤ .001. Thus, the faster participants inflated the balloons, the better their task performance. Interestingly, this effect was driven by significant correlations under stress, *r* = -.52, *p* ≤ .001, but not when participants had no stress, *r* = -.28, *p* = .083.

High and low socially anxious individuals did not differ in their speed-accuracy trade-off without stress, *z* = -0.67, *p* = .25 (*r* = -.30, *p* = .018 vs. *r* = -.41, *p* ≤ .001) or under stress, *z* = -0.98, *p* = .163 (*r* = -.51, *p* ≤ .001 vs. *r* = -.37, *p* = .002). The same relationship was true within the pool of high socially anxious individuals between the stress and no-stress condition and low socially anxious individuals between stress and no stress, all *z*s ≤ 1.43, all *p*s ≥.077.

To conclude, stress affected the speed-accuracy trade-off of high and low socially anxious individuals equally. Only under stress, individuals pumped faster and showed a heightened task performance.
