# Supplementary material for: Stress Makes the Difference: Social Stress and Social Anxiety in Decision-Making Under Uncertainty
Source: Front Psychol. 2021 Feb 22;12:578293. doi: 10.3389/fpsyg.2021.578293 (PMC7937725; doi:10.3389/fpsyg.2021.578293)
Supplement: Supplementary file 2 [file Data_Sheet_2.docx]

Supplementary Material

# Supplementary Tables

Table S1. *State anxiety ratings before and after the stress induction.*

|  | **No stress** | | **Stress** | | | |  |
| --- | --- | --- | --- | --- | --- | --- | --- |
|  | High socially anxious | Low socially anxious | High socially anxious | Low socially  anxious | |  |  |
| Time point |  |  |  | |  | | |
| *Pre* | 35.05 *(6.79)* | 33.62 *(6.25)* | 43.82 *(10.32)* | | 32.96 *(6.17)* |  |  |
| *Post* | 39.00 *(10.72)* | 36.76 *(10.43)* | 45.68 *(10.10)* | | 34.88 *(8.85)* |  |  |

*Note.* Means and standard deviations separately for high and low socially anxious individuals.

Table S2. *Ratings of the used emotional face expressions before the eBART.*

|  | **No stress** | | | | | | **Stress** | | | | | |  |  |
| --- | --- | --- | --- | --- | --- | --- | --- | --- | --- | --- | --- | --- | --- | --- |
|  | High socially anxious | | | Low socially anxious | | | High socially anxious | | Low socially anxious | | |  |  |  |
|  | Angry | Calm | Angry | | Calm | Angry | | Calm | Angry | Calm | | | |  |
| Dimension |  | | |  | | |  | |  | | | | | |
| *Valence* | 6.61 *(1.44)* | 3.95 *(1.23)* | 6.23 *(1.18)* | | 3.63 *(1.04)* | 6.17 *(1.48)* | | 3.84 *(1.36)* | 5.57 *(1.31)* | 3.57 *(1.54)* |  |  |  |  |
| *Arousal* | 5.69 *(1.73)* | 3.08 *(1.18)* | 4.78 *(1.59)* | | 2.39 *(0.98)* | 4.70 *(1.71)* | | 2.48 *(1.27)* | 3.66 *(2.16)* | 2.07 *(1.75)* |  |  |  |  |
| *Intensity* | 6.99 *(1.23)* | 3.80 *(1.34)* | 7.08 *(0.98)* | | 3.31 *(1.06)* | 7.25 *(0.84)* | | 3.24 *(1.15)* | 6.57 *(1.36)* | 2.79 *(1.18)* |  |  |  |  |

*Note.* Means and standard deviations separately for high and low socially anxious individuals.

| Table S3. *Risk avoidance behavior in the eBART.* | **No stress** | | | | | | **Stress** | | | | | |
| --- | --- | --- | --- | --- | --- | --- | --- | --- | --- | --- | --- | --- |
|  | *Fear-driven risk avoidance* | | | | | | | | | | | |
|  | High socially anxious | | | Low socially anxious | | | High socially anxious | | | Low socially anxious | | |
| Contingency | Pumps | Earn | Time | Pumps | Earn | Time | Pumps | Earn | Time | Pumps | Earn | Time |
| 100% | 39.00 *(17.38)* | 17.91 *(5.76)* | 0.35 *(0.21)* | 37.41 *(19.90)* | 16.53 *(6.01)* | 0.34 *(0.20)* | 34.24 *(14.90)* | 17.64 *(6.43)* | 0.36 *(0.16)* | 46.79 *(18.71)* | 19.22 *(5.99)* | 0.26 *(0.99)* |
| 50% | 37.52 *(16.75)* | 17.76 *(6.41)* | 0.34 *(0.18)* | 38.73 *(18.29)* | 16.74 *(5.63)* | 0.31 *(0.11)* | 37.35 *(15.92)* | 16.43 *(5.13)* | 0.36 *(0.17)* | 49.79 *(14.40)* | 19.76 *(4.59)* | 0.25 *(0.10)* |
| 0% | 37.01 *(16.31)* | 15.65 *(4.12)* | 0.35 *(0.18)* | 36.75 *(14.31)* | 16.29 *(4.73)* | 0.30 *(0.11)* | 38.23 *(16.26)* | 15.64 *(5.19)* | 0.34 *(0.13)* | 51.59 *(13.33)* | 20.84 *(6.64)* | 0.25 *(0.12)* |
|  | **No stress** | | | | | | **Stress** | | | | | |
|  | *Risk avoidance across Trials* | | | | | | | | | | | |
|  | High socially anxious | | | Low socially anxious | | | High socially anxious | | | Low socially anxious | | |
| Block | Pumps | Earn | Time | Pumps | Earn | Time | Pumps | Earn | Time | Pumps | Earn | Time |
| 1 | 34.25 (17.85) | 15.31 (5.34) | 0.45 (0.23) | 34.73 (17.10) | 16.80 (6.02) | 0.41 (0.22) | 31.79 *(14.95)* | 15.48 *(6.55)* | 0.42 *(0.17)* | 43.19 *(18.22)* | 18.66 *(4.86)* | 0.32 *(0.17)* |
| 2 | 37.02 (17.65) | 15.04 (4.55) | 0.31 (0.17) | 40.18 (21.17) | 16.21 (5.41) | 0.28 (0.10) | 37.79 *(14.90)* | 17.27 *(4.91)* | 0.33 *(0.14)* | 52.18 *(15.78)* | 20.94 *(4.94)* | 0.24 *(0.09)* |
| 3 | 37.08 (16.02) | 20.98 (6.18) | 0.29 (0.21) | 37.08 (16.02) | 17.21 (5.25) | 0.28 (0.11) | 38.00 *(14.54)* | 16.97 *(4.05)* | 0.31 *(0.16)* | 54.12 *(15.08)* | 20.22 *(6.81)* | 0.20 *(0.06)* |

*Note.* Values (and standard deviations) separately for high and low socially anxious individuals for risk avoidance across trials and fear-driven risk avoidance. n = Number of participants; Pumps = averaged adjusted number of pumps. Earn = sum of earned money in €; Time =averaged reaction times per pump in s.

Table S4. *Risk estimates in the REQ and RNOQ.*

|  | **Stress** | | **No Stress** | |
| --- | --- | --- | --- | --- |
|  | High socially anxious | Low socially anxious | High socially anxious | Low socially anxious |
| Risk estimates |  |  |  |  |
| *Social encounters* | 77.64 (36.99) | 91.64 (23.25) | 91.47 (19.33) | 99.71 (7.64) |
| *Socially negative outcomes* | 19.86 (15.02) | 10.60 (5.59) | 23.68 (8.67) | 14.81 (6.31) |

*Note.* Sum scores (and standard deviations) of the Risk of encounter social events (REQ) and the Risk of negative outcomes of such events (RNOQ) separately for high and low socially anxious individuals in the stress and non-stress condition.

# Supplementary Figures

**Fig. S1.** Means of angry and calm face rating at the baseline. Error bars reflect the standard error of means**.**
